# Sources, incidence and effects of non‐physical workplace violence against nurses in Ghana

**DOI:** 10.1002/nop2.43

**Published:** 2016-01-10

**Authors:** Isaac Mensah Boafo, Peter Hancock, Eyal Gringart

**Affiliations:** ^1^ School of Psychology and Social ScienceEdith Cowan University270 Joondalup DriveJoondalup 6027Western AustraliaAustralia

**Keywords:** Ghana, nurses, patient violence, sexual harassment, verbal abuse, workplace violence

## Abstract

**Aim:**

To document the incidence, sources and effects of workplace verbal abuse and sexual harassment against Ghanaian nurses.

**Methods:**

A cross‐sectional study was conducted in Ghana from 2013–2014 which surveyed 592 professional nurses and midwives working in public hospitals in Ghana using the health sector violence questionnaire.

**Results:**

The majority of participants were females (80%). The average age of participants was 31·76 years and the average number of years practising as nurse was 7·38. Twelve per cent of the participants experienced at least one incident of sexual harassment and 52·2% were exposed to verbal abuse. The majority of perpetrators of sexual harassment were medical doctors (50%). Relatives of patients emerged as the most frequent verbal abusers (45·5%). Chi‐square test showed statistically significant associations between gender and workplace violence and between workplace violence and intention to quit the nursing profession. The effects of workplace violence ranged from having disturbing memories about the incident to being ‘super alert’ and vigilant.

Establishing the incidence of workplace violence is a necessary step towards addressing the problem. It is concluded that educational programs must be designed for healthcare workers and the general public to foster awareness of the effects of workplace violence. Clear policies must also be instituted to address the problem.

## Introduction

Several studies have established that workplace violence is a problem for nurses internationally (Franz *et al*. 2010, Pinar & Ucmak 2011, Samir *et al*. 2012). However, there is a paucity of research on this issue in Africa. This paper, therefore, provides an empirical contribution from the global south, which adds to the broader discourse on the problems and challenges of nurses, specifically, the problems of verbal and sexual violence against them.

The current paper shows that workplace verbal abuse and sexual harassment constituted a major problem for nurses and health care in Ghana generally. Medical doctors were the most frequent perpetrators of sexual harassment against nurses. We argue that the high tolerance of Ghanaian society towards sexual harassment, hegemonic gender norms, the hierarchical structure of hospitals and lack of clear policies on workplace violence and harassment account for the comparatively higher rate of workplace sexual harassment against nurses in Ghana.

The region where nurses worked was found to impact on their exposure to verbal abuse. Nurses in the Greater Accra region constituted a majority of those who were abused verbally at the workplace. We suggest that the urbanized nature of the region, overcrowding in its hospitals, inadequate staff and poor infrastructure lead to frustration and dissatisfaction of patients and their relatives. This in turn increases their (patients and their relatives) tendency to use verbal abuse as a means of expressing their frustration and dissatisfaction. The results presented in this paper may be generalizable to the general population of nurses working in public hospitals in Ghana and to other nations.

### Background

While healthcare workers are generally recognized as increasing targets of violence, nurses are particularly at risk. In the UK, for instance, nursing has been described as the most dangerous occupation because of workplace violence (Elston *et al*. 2006). In a survey of licensed registered nurses and licensed practical nurses in Minnesota, USA, Gerberich *et al*. (2004) reported high rates of both physical and non‐physical violence against nurses. In another survey conducted among nurses and healthcare workers from two nursing homes, a psychiatric clinic and a disability workshop in Germany, Franz *et al*. (2010) reported that 70·7% of the respondents had experienced physical aggression, 89·9% experienced verbal abuse and 20·7% had been sexually harassed in the 12 months prior to the study (*N* = 123, RR = 38·8%).

Although various forms of workplace violence are often perpetrated against nurses, the prevalence and effects of non‐physical violence have long been underestimated. However, in the past decade, they gained attention to form a topical issue (Di Martino 2002a). Non‐physical violence, especially verbal abuse and sexual harassment, is a major problem facing both developed and developing nations with studies from Germany (Franz *et al*. 2010), Turkey (Çelik & Çelik 2007), Switzerland (Hahn *et al*. 2010), US (Gerberich *et al*. 2004), Hong Kong (Kwok *et al*. 2006), Jordan (AbuAlRub & Al‐Asmar, 2014) and Iran (Esmaeilpour *et al*. 2011) reporting high rates of verbal abuse and sexual harassment against nurses.

Regarding perpetrators of violence against nurses, the findings from the literature are congruent, they are usually patients, patients’ relatives, physicians, nurses themselves and other healthcare staff (AbuAlRub & Al‐Asmar 2014, Çelik & Çelik 2007, Hahn *et al*. 2010, Natan *et al*. 2011, Samir *et al*. 2012). Identification of the main perpetrators of violence is important for the formulation of context‐specific measures to address the problem, hence the need for this study in Ghana.

The impact of non‐physical violence can be greater than that of physical violence (Blanchar 2011). Many studies have documented the adverse effects of non‐physical workplace violence on nurses to the extent of them leaving their jobs (Gerberich *et al*. 2004, Jackson *et al*. 2002, Rippon 2000, Thomson 1997). AbuAlRub and Al‐Asmar (2014) reported several effects of verbal abuse on nurses akin to symptoms of post‐traumatic stress disorder and Çelik and Çelik (2007), reported that participants in their study had disturbed mental health function, decline in job performance and headache as a result of exposure to sexual harassment.

In Ghana, although there have been numerous studies that reported violence against women (Amoakohene 2004, GSS *et al*. 2009, Osam 2004), to our best of knowledge, there is no published work on the incidence of violence against nurses in the country. However, media reports suggest that violence against healthcare professionals is widespread (Ghana News Agency 2010, 2011, GhanaWeb 2001, Modern Ghana 2001).

Meanwhile, for any concrete policy or legislation to be put in place, there is the need for evidence on the magnitude of the problem. According to the International Council of Nurses (2000), the failure of governments to collect data on the incidence and frequency of workplace violence against nurses and to furnish policy‐makers with evidence‐based information account for the inability to address the problem. This paper thus provides empirical evidence as well as data on the incidence, sources and effects of non‐physical workplace violence against Ghanaian nurses. These data can be used by healthcare managers and policy‐makers towards addressing the problem. This study, therefore, aims at establishing the incidence of verbal abuse and sexual harassment against Ghanian nurses; and how nurses respond to these forms of workplace violence. It also examines the effects of verbal abuse and sexual harassment on Ghanaian nurses' intention to quit the nursing profession.

The current paper adopted the World Health Organisation's (WHO) definition for workplace violence. It defined workplace violence as ‘incidents where staff are abused, threatened, or assaulted in circumstances related to their work… involving an explicit or implicit challenge to their safety, well‐being or health’ (Wang *et al*. 2008 p. 31). Sexual harassment is defined as any unwanted, unreciprocated and unwelcome behaviour of a sexual nature that is offensive to the person involved and causes that person to be threatened, humiliated or embarrassed. Verbal abuse is the intentional use of language that humiliates degrades or indicates a lack of respect for the dignity and worth of an individual that creates fear, intimidation and anger in the nurse.

## Methods

A cross‐sectional descriptive questionnaire survey was conducted between September 2013 – April 2014 in 12 hospitals in Ghana comprising of two teaching hospitals, five regional and five district hospitals. In all, there are three teaching hospitals, nine regional hospitals and over a 100 district hospitals in Ghana.

### Sampling – hospitals

This study employed a multi‐stage sampling technique. The first stage was the selection of regions where the study was carried out. Five of the 10 administrative regions of the country were purposively selected for the study. These were Northern, Ashanti, Greater Accra, Eastern and Volta. The reason for purposively selecting these regions was to ensure that they were representative of the entire country. It ensured that all three major ecological zones, namely, the coastal, forest and savannah zones were represented. It also ensured that the various social, cultural, economic and demographical characteristics of the entire country were captured. These factors can produce differential experiences for nurses working in public hospitals.

The second stage involved the selection of hospitals for the study. This involved the use of both purposive and simple random techniques. In each of the 10 administrative regions, there is a regional hospital with the exception of the Ashanti Region. The regional hospitals in the selected regions were automatically selected for the study. The regional hospitals serve the entire region and they are usually the largest hospitals in the regions. They take referrals from other hospitals in the region; and where a case is beyond their capabilities, it is referred to a teaching hospital. In the Ashanti region where according to the Ghana Health Service no hospital is designated as a regional hospital, the Suntreso Government Hospital which is located in the Kumasi metropolis was chosen (for the purposes of this study) to represent a regional hospital due to its location and the diversity of the people it serves. In addition, two of the three teaching hospitals in the country were selected for the study. To ensure that the sample was representative of the Northern and Southern divide of the country, the Korle Bu Teaching Hospital and Tamale Teaching Hospital located in the Greater Accra Region and the Northern Region respectively were purposively selected.

Finally, five district hospitals were randomly selected for the study. Data on the districts in Ghana were obtained from the Ghana Statistical Service (GSS 2012). In all, there were 216 districts spread across the ten regions of the country. The districts in each of the five selected regions were put in five separate boxes. Each of the four research assistants and the researcher picked one district from each of the five boxes. This resulted in the selection of five districts.

### Sampling – participants

To participate in the study, one had to be a professional nurse and should have practiced for at least 12 months. In each hospital, qualified nurses in the selected units/departments were selected to take part in the study through a simple random sampling. A total of 1021 professional nurses were invited to take part in the survey, of which 685 accepted to participate and 592 returned questionnaires were valid for statistical analyses. The findings presented in this paper may be generalizable to the general population of nurses working in public hospitals in Ghana due to the representativeness of the sample and perhaps to nurses in other nations.

### Instrument

The International Labour Organisation, Internal Council of Nurses, the World Health Organisation and the Public Services International's (ILO *et al*. 2003) health sector workplace violence questionnaire was adapted for the current paper. This questionnaire has been use in several studies across different countries such as Iran (Esmaeilpour *et al*. 2011), Jordan (AbuAlRub & Al‐Asmar 2014), Brazil, Lebanon, Portugal, Bulgaria and Thailand (Di Martino 2002b). In Africa, it has been used in South Africa (Steinman 2003) and Mozambique (Adam *et al*. 2003).

The adjusted questionnaire was reviewed by five professional nurses for face validity, clarity and sensitivity of items. Both verbal and sexual violence were single‐item scales and for that matter it was not possible to determine their reliabilities using Cronbach's alpha. In view of this, prior to the study, these items were tested on 20 nurses who were not part of the study on two occasions with a 2‐week interval. The test–retest correlation coefficients (Nagy 2002) for sexual and verbal violence were 1·00 and 0·90 respectively. Data were collected between September 2013 – April 2014. To ensure that nurses working on all shifts were equally likely to be sampled, the data collection took place between 12:00 pm and 9:00 pm. Data were collected by the corresponding author and four trained research assistants.

### Data analyses

The data were analysed by means of Statistical Package for Social Sciences (SPSS) version 20. Descriptive statistics were used to summarize the socio‐demographical and workplace characteristics of participants and to establish the incidence and sources of workplace violence – percentages were used for categorical and means and standard deviations for continuous variables. Chi‐square test was used to assess the differences in exposure to non‐physical violence among the study variables. It was also used to test the association between workplace violence and intention to quit the nursing profession. All statistical analyses were two‐tailed and conducted at p < 0·05.

### Ethical considerations

The study was approved by the Edith Cowan University Human Research Ethics Committee (HREC) and the Ghana Health Service Ethics Review Committee. Before the commencement of data collection, permission was also sought from the Medical Superintendent or Hospital Administrator (where appropriate) and the Head of Nursing Services of the hospital after explaining to them the purpose of the study. The aims of the study were explained to all participants and they were assured of confidentiality. Participants were not allowed to write their names or any other information that could be used to trace them on the questionnaire. They were also informed that participation in the study was voluntary and they could withdraw their participation at any point with no penalty. Agreeing to complete the questionnaire (which was anonymous) was taken to be informed consent.

## Results

### Demographical and workplace characteristics

The 592 nurses who took part in the study were drawn from hospitals located in five regions of the country. The Greater Accra Region had the highest number of participants with over 42·1% of participants. This was because the region had the highest number of nurse population in Ghana. The number of nurses working in the region was more than twice the number of nurses working in the Eastern Region (Ghana Health Service 2010). A total of 22·8% of participants were nurses in the Eastern Region. A little over a third (38·7%) of the participants worked in regional hospitals and a third (32·9%) worked in teaching hospitals.

The gender distribution of the sample reflected the general distribution of males and females in the nursing profession in Ghana with approximately 20% being males and 80% being females. The age of participants ranged from 21‐60 years with a mean age of 31·76 years. However, the mean age of male nurses was lower than that of females (mean = 28·89, sd <0·44 vs. mean = 32·51, sd 10·40). About two‐thirds (67·7%) of the participants fell within the age range of 21‐30 years. This gender difference in age could be the result of males staying in the nursing profession for a shorter time.

More than half of the sample (61·3%) had a Diploma qualification and 53% of the entire sample were staff nurses/midwives. The number of years participants reported working as a nurse ranged from 1‐40 years with a mean of 7·38 (sd 9·53). The mean number of years of working for male nurses was 3·74 (sd 4·12) and females was 8·33 (sd 10·28). An independent samples *t*‐test shown that the difference between males and females in terms of number of years of working experience was statistically significant, t (494·71) = 7·61, p = 0·001. This gender difference could be because males do not stay in the nursing for a long time due to the feminized nature of the profession. Of the entire sample, 90·1% were engaged in direct patient care with 17% of the sample working in the outpatient department (OPD) and 35·5% working at the medical and surgical units.

With regard to the presence of reporting procedures for workplace violence against nurses, 72·9% of nurses reported there were reporting procedures and 80·3% of those who reported there were procedures for reporting violence (57·8% of the entire sample) indicated they know how to use these procedures. However, as would be seen later in this paper, a significant number of participants who experienced violence did not report such incidents. There could be several plausible explanations for this, including fear of victimization and the belief that nothing would be done if reported.

Although the mean score for nurses’ concern over workplace violence suggests that nurses were moderately concerned about workplace violence, the mode revealed that many nurses were extremely concerned about workplace violence (min =1, max= 5, mean= 3·05, sd 1·51, mode = 5). Being concerned means that nurses did not feel safe in their workplaces as far violence is concerned. It is an admission that they are at risk of violence. Table 1 gives more information on the demographical and workplace characteristics of the participants.

**Table 1:** Socio‐demographical and workplace characteristics (*N *=* *592)

Item description	*n* (%)
Region	
Greater Accra	249 (42·1)
Eastern	130 (22·0)
Ashanti	76 (12·8)
Volta	51 (8·6)
Northern	86 (14·5)
Hospital type	
District hospital	168 (28·4)
Regional hospital	229 (38·7)
Teaching hospital	195 (32·9)
Gender	
Female	469 (79·2)
Male	123 (20·8)
Age group	
21‐30	398 (67·7)
31‐40	98 (16·7)
41‐50	37 (6·3)
51‐60	55 (9·4)
Marital status	
Single	310 (52·5)
Married	280 (47·5)
Educational attainment	
Certificate	79 (13·4)
Diploma	363 (61·7)
Bachelor's degree or higher	146 (24·8)
Units/Department	
Critical care	119 (20·2)
Outpatient department	100 (17·0)
Medical‐surgical unit	209 (35·5)
Special units	160 (27·2)
Position/Grade	
Staff nurse/midwife	308 (53·0)
Snr. staff nurse/midwife	132 (22·7)
Nursing/Midwifery officer	72 (12·4)
Snr nursing/midwifery officer	42 (7·2)
Principal nursing/midwifery officer	28 (4·8)
Are there procedures for reporting violence?	
Yes	427 (72·9)
No	159 (27·1)
If yes, do you know how to use the reporting procedures	
Yes	342 (80·3)
No	84 (19·7)
Age (min = 21, max = 60, mean = 31·76, sd 9·69)	
Number of years in nursing (min = 1, max = 40, mean = 7·38, sd 9·53)	
How concerned are you about violence (min = 1, max = 5, mean = 3·05, sd 1·51)	
How many other staff usually work with you at the same time? (mean 3·34, sd 2·01)	

### Incidence, distribution and sources of workplace sexual harassment

Seventy‐two of the 592 participants (12·2%) reported that they have been sexually harassed in their workplace in the past 12 months before the study. Of the 72 participants who were sexually harassed in the workplace, 83% reported that they were harassed inside the hospital and over 50% indicated that a medical doctor sexually harassed them. The second most commonly cited perpetrators of sexual harassment were relatives of patients.

More than two‐thirds of all the sexual harassment incidents were reported by nurses working in the Greater Accra Region. Examination of the regional differences with regard to the prevalence of sexual harassment using Chi‐square shows a statistically significant association between the region where a participant worked and the experience of sexual harassment (χ²= 26·82, df = 4, N = 591, p < 0·01) with 20·9% of nurses working in the Greater Accra Region, 9·2% in the Eastern Region, 5·9% in the Volta Region, 5·3% in the Ashanti Region and 3·5% in the Northern Region indicating they have been sexually harassed in their workplace. There were no statistically significant differences between hospital type and sexual harassment.

Over 90% of nurses who were sexually harassed in the workplace were females (*χ*²= 9·39, df = 1, N = 591, p < 0·01). A computation of the odds ratio revealed that female nurses were 3·9 times more likely than males to be victims of workplace sexual harassment (95% C.I.: 1·54 – 9·90). Age was also found to be significantly associated with being harassed sexually in the workplace (*χ*² = 11·31, df = 3, N = 587, p = 0·01). A negative relationship existed between age and being harassed sexually, with those in the youngest age group (21‐30) constituting over 80% of nurses who have experienced workplace sexual harassment. Correspondingly, marital status of participants was also significantly associated with being harassed in the workplace (*χ*² = 23·12, df = 3, N = 589, p < 0·01). Nurses who were not married experienced more sexual harassment (75%) than those who were married (25%).

The relationship between position and sexual harassment was also statistically significant (*χ*² = 10·85, df = 4, N = 581, p = 0·03). Staff nurses/midwives reported more cases of sexual harassment than any other position. Although there were differences in terms of prevalence rate across units, this was not statistically significant. Table 2 presents data on the prevalence and sources of workplace sexual harassment against nurses.

**Table 2:** Incidence, sources and distribution of non‐physical workplace violence

Item description	Verbal *n* (%)	Sexual *n* (%)
Region		
Greater Accra	153 (49·0)	50 (69·4)
Eastern	65 (20·8)	12 (16·7)
Ashanti	36 (11·5)	4 (5·6)
Volta	28 (9·0)	3 (4·2)
Northern	30 (9·6)	3 (4·2)
Hospital type		
District hospital	92 (29·5)	19 (26·4)
Regional hospital	119 (38·1)	28 (38·9)
Teaching hospital	101 (32·4)	25 (34·7)
Gender		
Female	259 (83·0)	67 (93·1)
Male	53 (17·0)	5 (6·9)
Age group		
21‐30	223 (71·9)	59 (81·9)
31‐40	49 (15·7)	11 (15·3)
41‐50	17 (5·4)	1 (1·4)
51‐60	21 (6·7)	1 (1·4)
Marital status		
Single	167 (53·9)	54 (75)
Married	143 (46·1)	18 (25)
Units departments		
Critical care	56 (17·9)	12 (16·7)
OPD	64 (20·5)	13 (18·1)
Medical‐surgical	112 (35·9)	32 (44·4)
Special units	80 (25·6)	15 (20·8)
Position/grade		
Staff nurse/midwife	159 (51·0)	47 (65·3)
Snr staff nurse/midwife	80 (25·6)	9 (12·5)
Nursing/midwifery officer	41 (13·1)	9 (12·5)
Snr nursing/midwifery officer	23 (7·4)	7 (9·7)
Principal nursing/midwifery officer	8 (2·6)	0 (0)
Where did the incident happen		
Inside the hospital	304 (98·4)	60 (84·7)
Outside the hospital	5 (1·6)	11 (15·3)
Perpetrator of last sexual harassment		
Patient	83 (26·6)	8 (11·3)
Patient's relations	142 (45·5)	13 (18·3)
Doctor	25 (8·0)	39 (54·9)
Nurse	52 (16·7)	5 (7·0)
Other staff	10 (3·2)	6 (8·5)

### Effects and reactions to workplace sexual harassment

Responses to a multiple‐response question revealed that participants reacted to workplace sexual harassment in a variety of ways. However, the most common reaction was to tell the perpetrator to stop (55·6%). It is interesting to note that of the 72 participants who were harassed sexually, only three (3) reported the incident to a senior staff member or ward in‐charge, however, a significant number (29) told a colleague about the incident. The analyses revealed that all three incidents, which were reported, were investigated.

Concerning the reasons why nurses did not report workplace sexual harassment, 50 (80·6%) were of the view that it was not important, 7(11·3%) perceived sexual harassment to be part of the job, 14 (22·6%) felt ashamed, 12 (19·4%) thought that no action would be taken if reported and 33 (53·2%) indicated they did not know who to report to. Not knowing who to report to may be a result of the fact that there are no specific procedures for reporting such incidents. Moreover most of the perpetrators of sexual harassment were doctors who are perceived by nurses to be their superiors, thereby heightening their belief that nothing would be done if reported. Reactions to workplace sexual harassment are presented in Table 3.

**Table 3:** Reactions to non‐physical workplace violence

Variable	Verbal *n* (%)	Sexual *n* (%)
Responses to violence		
Took no action	143 (46·3)	17 (23·6)
Told the person to stop	65 (21·0)	40 (55·6)
Told family/friends	18 (5·8)	6 (8·3)
Told a colleague	61 (19·7)	29 (40·3)
Sought transfer to another unit	4 (1·3)	1 (1·4)
Completed an incident form	6 (1·9)	1 (1·4)
Sought help from association	0 (0)	8 (11·1)
Tried to pretend it never happened	113 (36·6)	8 (11·1)
Tried to defend myself physically	0 (0)	34 (47·2)
Sought counselling	4 (1·3)	1 (1·4)
Reported to a senior staff/in‐charge	98 (31·7)	3 (4·2)
Pursued prosecution	1 (0·3)	1 (1·4)
Was action taken to investigate the incident? YES	58 (18·6)	3 (4·2)
Reasons for not reporting incidents		
It was not important	118 (62·8)	50 (80·6)
Such abuse is part of the job	104 (55·3)	7 (11·3)
I felt ashamed	6 (3·2)	14 (22·6)
I was afraid of negative consequences	6 (3·2)	2 (3·2)
No action will be taken if reported	79 (42·0)	12 (19·4)
Did not know whom to report to	7 (3·7)	33 (53·2)
Other reasons	5 (2·7)	6 (9·7)

Participants faced several problems and difficulties as a result of sexual harassment. More than one‐third (33·8%) of the nurses had repeated disturbing memories and thoughts about a sexual harassment incident. Close to half (46·4%) of the nurses had difficulties avoiding thinking about, or having feeling related to an incident of sexual harassment. A higher proportion (66·2%) reported being ‘super alert’ and ‘on guard’ following their harassment. Approximately, 17% actually became extremely ‘super alert’ and ‘on guard’. These are particularly remarkable findings considering that most perpetrators of sexual harassment were medical doctors. Nurses and medical doctors are required to work closely together to achieve optimum health outcomes. These symptoms, which are congruent with having experienced trauma, may strain the collaborative interaction between a doctor and a nurse. This may in turn impact on patient outcomes. Data on the effects of sexual harassment on participants are presented in Table 4.

**Table 4:** Effects of non‐physical workplace violence on Ghanaian nurses

Variable	Verbal *n* (%)	Sexual *n* (%)
Repeated disturbing memories, thoughts or images of the incident		
Not at all	146 (47·6)	47 (66·2)
A little bit	95 (30·9)	15 (21·1)
Moderately	38 (12·4)	7 (9·9)
Quite a bit	17 (5·5)	2 (2·8)
Extremely	11 (3·6)	0 (0)
Avoiding thinking or talking about the incident or having feeling related to it		
Not at all	109 (35·9)	38 (53·5)
A little bit	93 (30·6)	22 (31·0)
Moderately	45 (14·8)	5 (7·0)
Quite a bit	32 (10·5)	5 (7·0)
Extremely	25 (8·2)	1 (1·4)
Being ‘super alert’ or watchful and on guard		
Not at all	68 (22·8)	24 (33·8)
A little bit	69 (23·2)	20 (28·2)
Moderately	43 (14·4)	11 (15·5)
Quite a bit	51 (17·1)	4 (5·6)
Extremely	67 (22·5)	12 (16·9)

### Incidence, distribution and sources of workplace verbal abuse

The incidence of verbal abuse was higher than that of sexual harassment. Of the 592 nurses, 312 (52·7%) reported to have suffered verbal abuse in the 12 months preceding the study. Out of these, 259 (83·0) were female nurses and 53 (17·0%) were male nurses. Chi‐square test performed on gender and verbal abuse indicates that this difference was statistically significant (*χ²* = 5·98, df = 1, N = 590, p = 0·01). The cross‐tabulations reveal that 55·5% of female nurses have been victims of verbal abuse compared with 43·1% of male nurses.

With regard to regional distribution of verbal abuse, the Greater Accra region had the highest prevalence rate with 49% of all verbally abused nurses coming from that region. This is more than twice the proportion reported by nurses in the eastern region (20·8%), who happen to have the second highest prevalence rate for verbal abuse. Chi‐square test showed a statistically significant relationship between region, where nurses worked and being abused verbally (*χ²* = 20·24, df = 4, N = 590, p < 0·01).

No statistically significant association between hospital type and being abused verbally was found. Similarly, age was not statistically significantly associated with verbal abuse. Chi‐square analyses also showed no statistically significant association between units where nurses worked and experiencing workplace verbal abuse. The non‐significant association between unit and exposure to verbal abuse is an indication of the generalized nature of the situation. Verbal abuse is not concentrated in any one unit/department.

The findings also indicate that workplace verbal abuse reduced progressively with one's position on the occupational ladder. Nurses in higher positions and/or ranks reported lower rates of verbal abuse that were statistically significant (χ² = 9·75, df = 4, N = 580, p = 0·05). With regards to frequency of workplace verbal abuse, nearly three quarters (73·7%) of participants have been abused verbally more than once in the 12 months prior to the study. Relations of patients were found to be the most frequent source of workplace verbal abuse (45·5%), followed by patients (26·6%) and then other nurses (16·7%). Table 2 displays data on the prevalence and sources of workplace verbal abuse against nurses.

### Reactions to workplace verbal abuse

Of the 312 participants who were verbally abused, 46·3% took no action, about a fifth (19·7%) told a colleague and over a third (36·6%) tried to pretend it never happened. Whilst 98 (31·7%) participants indicated that they reported the incident, only 58 (18·6%) indicated that action was taken to investigate the causes of the reported verbal abuse.

Regarding consequences for the perpetrator, 11·9% indicated that a verbal warning was given and 2·2% reported a written warning was given. Only 22 (22·4%) reported to be satisfied with the manner in which, the incident was handled and 36·7% indicated that they were dissatisfied with the way it was handled.

Multiple‐response item examining the reasons for not reporting workplace verbal abuse showed that the majority of the participants did not report the incident, because they believed it was futile to do so (*n *=* *118; 62·8%). More than half (55·3%) of those who have been abused verbally in the course of their work reported that verbal abuse is part of the job. Data about reactions to workplace verbal violence are presented in Table 3.

The problems and difficulties reported by the participants as a result of verbal abuse incidents were: (a) 38 of the 312 participants (12·2%) who had been verbally abused reported that they were moderately bothered by repeated, disturbing memories, thoughts or images of the abuse; (b) 14·4% had been bothered moderately by avoiding talking about the abuse or avoiding having feelings related to it; (c) 21·5% had been extremely ‘super‐alert’ (hyper‐vigilant) and on guard. Table 4 displays the effects of workplace verbal abuse on nurses.

### Non‐physical workplace violence and intention to quit

The current paper also investigated the association between non‐physical workplace violence and intention to quit the nursing profession using chi‐square. The analyses revealed a statistically significant association between verbal abuse and intention to quit the nursing profession (χ² = 14·42, d.f. = 1, *N* = 583, *P* < 0·05). However, the association between sexual harassment and intention to quit the nursing profession was not statistically significant.

As shown in Table 3, more than half of the sample believed that verbal abuse is ‘part of the job’ and close to two‐thirds (62·8%) considered reporting as ‘not important’. In view of this, the association between verbal abuse and intention to quit could be as a result of nurses thinking that the only way to combat the problem is to leave the profession.

## Discussion

This paper adds to the body of literature supporting the argument that nurses are exposed to greater abuse in their workplaces and that workplace violence has detrimental effects on both nurses and the health care system (AbuAlRub & Al‐Asmar 2014, Gerberich *et al*. 2004). An incident rate of 52·7% was found for verbal abuse in our study. This paper is consistent with the literature (AbuAlRub & Al‐Asmar 2014, Franz *et al*. 2010, Natan *et al*. 2011) as patients and their relatives constituted the main perpetrators of verbal abuse against nurses.

A statistically significant association was found between region where participants worked and the experience of verbal abuse. Nurses in the Greater Accra region reported significantly higher incidents of verbal abuse episodes. Several plausible factors can explain this higher exposure of nurses in that region to verbal abuse. These include overcrowding of the hospitals, inadequate staff and infrastructure, leading to long waiting times and low quality service. The frustration that patients and their relatives may have to go through before they are attended to (due to long waiting times) and dissatisfaction with service could make them more inclined to abuse nurses verbally. Although almost all Ghanaian hospitals are known to be under‐resourced (Pillinger 2011), the impact of this is most felt in the most urbanized parts of the country such as Accra. This is an avenue for further study.

With regard to workplace sexual harassment, an incidence rate of 12·2% was reported. This rate is higher than what has been reported in other African studies (Steinman 2003, Adam *et al*. 2003, Samir *et al*. 2012). We found that gender, age, marital status and region of work were statistically significantly associated with sexual harassment. The majority (54·9%) of sexual harassment incidents were perpetrated by physicians. This finding is consistent with previous studies (Çelik & Çelik 2007, Subedi *et al*. 2013). Çelik and Çelik (2007), for instance, found in their Turkish study that over 50% of sexual harassment episodes were perpetrated by physicians. This is, however, inconsistent with that of Kwok *et al*. (2006) who reported that patients and their relatives are the most frequent perpetrators of sexual harassment. The findings in the current paper also support the argument that young, unmarried women living in urban locales and occupying lower status positions are the most frequent targets of sexual harassment (Gullotta & Bloom 2003, Subedi *et al*. 2013) considering the fact that the majority of physicians in Ghana are males and they wield or are perceived to wield much more power than nurses in the hospital.

With regard to sexual harassment, other socio‐cultural factors such as patriarchal gender relations may play a role (Amoakohene 2004, Nukunya 1992). It is also worth stating that, in Ghana, the local slang for approaching and courting a woman is ‘chasing’, which implies an element of aggression, no matter how friendly this may be perceived by the ‘chaser’ or the ‘chased’. ‘For some determined chasers, harassment may then be seen as a necessary strategy in chasing to persuade a girl/woman to succumb to persistent advances’ (Ayeetey 2004 p. 31). This societal acceptance of sexual harassment may explain why a significant number of victims did not report such acts to the nursing and or hospital authorities. This paper therefore argues that, in the absence of clear policies prohibiting sexual harassment in the workplace, these contextual factors may provide a conducive atmosphere for the harassment of nurses sexually by medical doctors.

The current paper found that nurses do not readily report non‐physical violence against them. A multiple‐response item showed that the reasons for this included the perception that such abuse is ‘part of the job’, or ‘not important to report’, or ‘no action will be taken if reported’ and ‘not knowing whom to report to’. This finding is also corroborated by other studies such as AbuAlRub and Al‐Asmar (2014) and Natan, Hanukayev and Fares (Natan *et al*. 2011). A study by Natan and colleagues, suggested that the failure of nursing and hospital administrations to act on reported cases of violence partly accounted for the failure of nurses to report violent incidents.

Both sexual and verbal workplace violence had an impact on the psychological well‐being of nurses. Sexual harassment and verbal abuse affected the way nurses approached their jobs subsequently. We found that 34·2% of those who experienced verbal abuse and 40% of those who suffered sexual harassment reported being moderately to extremely ‘super alert’ and ‘on guard’ following their experience. This is a critical finding as the major actors in health care, nurses, physicians, patients and their relatives must work together to achieve optimum care. Sexual harassment from physicians may strain the working relationship between them and their targets. We also found that workplace verbal abuse is significantly associated with intention quit the nursing profession. This finding is consistent with other studies, which have reported that nurses quitted their jobs or had the intention to quit as a result of workplace violence (Gerberich *et al*. 2004, Oulton 2006, Shahzad & Malik 2014). The desire of nurses in the current paper to quit the nursing profession subsequent to the experience of verbal abuse could be because many nurses consider it to be part of the job and for that matter the only way to combat the abuse is to leave the profession. It can also be explained through the association between workplace violence and job satisfaction (Alam & Mohammad 2010, Coomber & Barriball 2007). This study thus confirms that non‐physical workplace violence can have an equally severe (if not more) impact in this regard as physical violence.

The belief that verbal abuse is ‘a part of the job’ could also account for the reason why many nurses considered reporting such abuse as ‘not important’. Several studies have reported that many nurses do not report abuse against them (Ferns 2006, Shahzad & Malik 2014). Shahzad and Malik (2014) found that about 80% of nurses did not report violence against them in the workplace. Natan *et al*. (2011) in their study of Israeli nurses suggested that nurses considered reporting workplace violence unimportant because no action is taken when such incidents are reported. The research presented in this paper suggests it should be taken seriously as nursing is a key profession in any society.

### Limitations

This study was conducted among hospital‐based nurses and does not include nurses in clinics and health posts located in rural and or remote areas of the country. It also concentrated on nurses in government hospitals and thus excluded those in private hospitals. This means that the findings of this study may not be generalizable onto this section of the nurses’ population in Ghana and elsewhere. The cross‐sectional nature of the study means that no causal time‐related effects can be drawn from it.

## Conclusion

This paper constitutes the very first study to be conducted on the incidence of workplace verbal abuse and sexual harassment against nurses in Ghana. The findings in this paper show that workplace verbal abuse and sexual harassment are major problems for nurses. More than half of the sample (52·7%) were exposed to verbal abuse and 12·2% were sexually harassed at the workplace in the 12 months preceding the study. Gender was statistically significantly associated with verbal abuse and sexual harassment. Participants who worked at the Greater Accra region experienced higher rates of sexual harassment and verbal abuse. Both forms of violence had significant impacts on the psychological well‐being of participants. Furthermore, verbal abuse was statistically significantly associated with intention to quit the nursing profession. Despite these, a significant number of nurses did not report verbal abuse and sexual harassment against them. We found that young unmarried nurses were the most frequent targets of sexual harassment. The most frequent perpetrators of sexual harassment was found to be medical doctors and for verbal abuse, relatives of patients. It was argued that the hierarchical structure of the hospitals, hegemonic gender relations and the high tolerance of the general society to sexual harassment explain the high incidence and non‐reporting of the phenomenon.

### Implications for interventions and practice

Further research is needed to compare the incidence of non‐physical workplace violence against nurses in the private and public health sector. There is also the need for further research to assess a wider range of effects of violence against nurses. This study established an association between verbal abuse and intention to quit. There is the need for longitudinal studies to establish whether Ghanaian nurses who have the intention to quit actually quit the profession. That is to say there is the need to find out whether intentions lead to actual turnover among Ghanaian nurses.

With regard to dealing with the problem of verbal abuse and sexual harassment against Ghanaian nurses, there should be clear policies on workplace violence in the healthcare system. In the absence of clear policies, which will make perpetrators aware of the effects, workplace verbal abuse and sexual harassment is likely to continue and may escalate. Educational programs could be designed for health care providers, patients and the general public that foster awareness of the phenomenon of workplace violence. Different mass communication methods could be used in enhancing awareness of the public on the adverse effects of workplace violence and how the entire society suffers as a result of that. Nurses should also be encouraged to report every violent act against them promptly and such reports must be acted on immediately so that nurses come to understand that such reporting important and is not futile. Finally, teaching universities may need to amend curricula to ensure doctors and nurses are more aware of the issues reported in the current paper and are trained to avoid and deal with, negative behaviours.

## Author contributions

All authors have agreed on the final version and meet at least one of the following criteria [recommended by the ICMJE (http://www.icmje.org/recommendations/)]:

- substantial contributions to conception and design, acquisition of data, or analysis and interpretation of data;
- drafting the article or revising it critically for important intellectual content.
